# Comparative Physical Mapping of 18S rDNA in True Bug Species of the Families Gerridae and Mesoveliidae: First Data on the Semiaquatic Infraorder Gerromorpha (Heteroptera, Hemiptera)

**DOI:** 10.3390/biology15131075

**Published:** 2026-07-05

**Authors:** Natalia Golub, Boris Anokhin, Desislava Stoianova, Valentina Kuznetsova

**Affiliations:** 1 Zoological Institute, Russian Academy of Sciences, Unverisitetskaya Emb. 1, 199034 St. Petersburg, Russia; psocids@zin.ru; 2 Institute of Biodiversity and Ecosystem Research, Bulgarian Academy of Sciences, 1000 Sofia, Bulgaria; d.st.stoianova@gmail.com

**Keywords:** karyotype, FISH, ribosomal DNA (rDNA), Gerridae, Mesoveliidae, Gerromorpha, Heteroptera

## Abstract

**(1) Background:** Over the past few decades, the number and chromosomal arrangement of rDNA loci have been successfully used as cytogenetic markers in comparative studies of many insect taxa, including the hemipteran suborder Heteroptera (or true bugs). Of the seven generally accepted infraorders of true bugs, such data are available only for three: one of the early branching infraorders Nepomorpha and the sister infraorders Cimicomorpha and Pentatomomorpha. To address this gap, we obtained rDNA-FISH data for another “early” evolutionary lineage of true bugs, the infraorder Gerromorpha, in which we studied four species belonging to the families Gerridae and Mesoveliidae. **(2) Methods:** Standard karyotypes were studied using the Schiff–Giemsa method. To study the chromosomal distribution of the major rDNA (45S rDNA), the Fluorescence In Situ Hybridization (FISH) method was used with an 18S rDNA probe. **(3) Results:** Three variants of the chromosomal arrangement of 18S rDNA were identified in the studied species. The rDNA loci were located on one pair of autosomes in *Aquarius paludum* (n = 11AA + X) and *Gerris lacustris* (n = 10AA + X); on one pair of autosomes and on the X chromosome in *Limnoporus rufoscutellatus* (n = 10AA + X) (Gerridae); on one of the four X chromosomes and on the Y chromosome in *Mesovelia furcata* (n = 15AA + X_1_X_2_X_3_X_4_Y) (Mesoveliidae). The karyotype of *L. rufoscutellatus* was studied for the first time. **(4) Conclusions:** Our study provides the first data on the rDNA-FISH location for the infraorder Gerromorpha and expands current understanding of the chromosomal distribution of rDNA arrays in Heteroptera as a whole.

## Simple Summary

We used the Fluorescence In Situ Hybridization (FISH) technique to determine the genomic distribution of 18S rDNA arrays in *Aquarius paludum* (n = 11AA + X), *Gerris lacustris* (n = 10AA + X) and *Limnoporus rufoscutellatus* (n = 10AA + X), belonging to the family Gerridae, and in *Mesovelia furcata* (n = 15AA + X_1_X_2_X_3_X_4_Y), belonging to the family Mesoveliidae (Gerromorpha, or semiaquatic bugs). The karyotype of *L. rufoscutellatus* was studied here for the first time. We have shown that the studied species have one or two rDNA sites (loci) per haploid genome, which is typical for most true bugs. The rDNA loci were located in different ways: on one pair of autosomes in *G. lacustris* and *A. paludum*; on one pair of autosomes and on the X chromosome in *L. rufoscutellatus*; on one of the four X chromosomes and on the Y chromosome in *M. furcata*. The obtained rDNA-FISH data are the first for the entire infraorder Gerromorpha.

## 1. Introduction

The true bugs (Hemiptera: Heteroptera) are one of the most diverse taxa among non-holometabolous insects. The suborder Heteroptera comprises over 40,000 described species in about 100 families classified into seven infraorders, including Enicocephalomorpha, Dipsocoromorpha, Gerromorpha, Leptopodomorpha, Nepomorpha, Cimicomorpha and Pentatomomorpha [1,2]. The infraorder Gerromorpha Popov, 1971 (or semiaquatic bugs), numbering about 2100 described species distributed worldwide (with the exception of Antarctica), is a relatively small but remarkably diverse taxon of Heteroptera in terms of morphology, phylogeny, ecology, behavior, adaptation, and fossil history [2,3,4,5].

Despite the great interest in Gerromorpha and the presence of a significant body of literature on the unique characteristics of semiaquatic bugs, the available information on their cytogenetics is scarce and limited by the number of chromosomes, sex chromosome systems, and some features of meiosis [6,7,8]. Modern methods of molecular cytogenetics have long been successfully used in chromosome studies of true bugs (e.g., [9,10,11,12,13,14,15,16,17,18,19,20,21,22,23,24,25,26]). However, so far, there has not been a single attempt to use these methods to study Gerromorpha.

Currently, eight families (in four superfamilies) are recognized in Gerromorpha: Hebridae (Hebroidea), Hydrometridae, Paraphrynoveliidae, Macroveliidae (Hydrometroiodea), Hermatobatidae, Veliidae, Gerridae (Gerroidea), and Mesoveliidae (Mesoveloidea) [27,28]. Our article will focus on the families Gerridae (commonly known as water striders, although also as pond-skaters, water skitters, water gliders, see skaters, magic bugs or Jesus bugs) and Mesoveliidae (commonly known as water traders).

The Gerridae Leach, 1815 is one of the most diverse families of Gerromorpha in terms of morphology, biology, behavior, life history, ecology, feeding strategies and reproductive biology. Due to some specific and even intriguing features, water striders attract the attention of specialists of various profiles, and therefore, they are the most famous representatives of gerromorphs. They inhabit a wide range of freshwater, brackish and even marine habitats, and are clearly adapted to live on the surface of the water, where they “‘skate’ and/or ‘jump-and-slide’ in a very characteristic way” [2,3,29,30,31,32]. Gerridae comprise more than 800 extant species assigned to at least 71 genera, eight subfamilies and eight tribes [2,5,33]. However, a recent comprehensive molecular analysis of the family, including representatives of all traditionally recognized subfamilies and tribes, has shown that the existing classifications do not fully reflect the evolutionary history of this taxon [5]. Within Gerridae, the pond skaters of the subfamily Gerrinae are among the best-known and most frequently encountered semi-aquatic bugs [3]. In northern temperate regions, most species of water striders belong to three genera of the Gerrinae: *Gerris* Fabricius, 1794, with about 43 species distributed in Holarctic and Nearctic, cosmopolitan genus *Aquarius* Schellenberg, 1800, with 19 species, and Holarctic genus *Limnoporus* Stål, 1868, with seven described species [34]. It is important to note that until very recently, most water strider species from the Northern Hemisphere were classified within a single genus, *Gerris*, with the subgenera *Aquarius*, *Limnoporus*, and *Gerris* s.str., which were subsequently restored as genera (see [35] for references).

The Mesoveliidae Douglas & Scott, 1867 is a relatively small group comprising 12 genera and approximately 50 described species [2,28]. Within this family, the genus *Mesovelia* Mulsant & Rey, 1852 ranks first in terms of species richness, numbering 27 species that have a noticeable cosmopolitan distribution all over the world [2].

The chromosomes of Gerromorpha, like those of all Heteroptera, are holokinetic [6], meaning that they lack localized centromeres, and the only criterion for identifying individual chromosomes in the karyotype is their size, if there are any size differences at all. The search for chromosomal markers is therefore an important task for comparative analysis of karyotypes. In this study, we used molecular cytogenetics for the first time to identify chromosomal markers in semiaquatic bugs by mapping 18S rDNA through FISH on the chromosomes of three species of Gerridae, belonging to the above-mentioned principal northern temperate genera *Gerris*, *Aquarius* and *Limnoporus* (Gerrinae), as well as *Mesovelia furcata* Mulsant & Rey, 1852 from the family Mesoveliidae.

## 2. Materials and Methods

The material used for chromosomal analysis is listed in Table 1.

Only males were used for chromosome analysis. The specimens were anesthetized and then fixed in the field in a 3:1 Carnoy’s solution (96% ethanol: glacial acetic acid) and stored at 4 °C. In the laboratory, testes were dissected out in a drop of 45% acetic acid and squashed on the slide. The cover slips were removed using the dry ice technique [36].

The Schiff–Giemsa method was used to study standard karyotypes in accordance with the protocol published by Grozeva and Nokkala (1996) [37]. The slides were treated in 1N HCl at room temperature for 20 min, hydrolyzed in 1N HCl at 60 °C for 8 min, stained with Schiff’s reagent for 20 min, rinsed successively in distilled water and Sorensen’s phosphate buffer, pH 6.8. Then, the slides were stained with a 5% Giemsa solution in the same buffer for 20–30 min. After staining, the slides were briefly rinsed with distilled water, air-dried, and mounted in Entellan (Merck KGaA, Darmstadt, Germany).

Chromosomal mapping of 18S rDNA clusters was performed in accordance with a previously published protocol [38]. Briefly, the target 18S rDNA probe (about 1200 bp fragment) was amplified via polymerase chain reaction (PCR) and labeled with biotin-11-dUTP (Fermentas, EU) using specific primers: 18S_F (5′-GATCCTGCCAGTAGTCATATG-3′) and 18S_R (5′-GAGTCAAATTAAGCCGCAGG-3′). Genomic DNA was extracted from the true bug *Pyrrhocoris apterus* (Linnaeus, 1758) using the standard CTAB extraction method [38]. An initial denaturation period of 3 min at 94 °C was followed by 35 cycles of 30 s at 94 °C, annealing for 30 s at 53 °C, and extension for 110 s at 72 °C, with a final extension step of 3 min at 72 °C. The chromosome preparations were treated with 100 g/mL RNAse A and 5 mg/mL pepsin solution to remove excess RNA and proteins. Chromosomes were denatured in the hybridization mixture containing labeled 18S rDNA probe with the addition of salmon sperm blocking reagent and then hybridized for 42 h at 37 °C. The 18S rDNA probe was detected with NeutrAvidin-Fluorescein conjugate (Invitrogen, Karlsbad, CA, USA). The chromosomes were mounted in an antifade medium (ProLong Gold antifade reagent with DAPI, Invitrogen) and covered with a glass coverslip. From 10 to 30 chromosome plates were examined on each slide. The most informative plates were selected for imaging.

The slides subjected to standard staining and FISH were photographed under oil immersion (X100 objective) using a Leica DM 6000 B microscope, Leica DFC 345 FX camera, and Leica Application Suite 4.5.0 software with an Image Overlay module (Leica Microsystems, Wetzlar, Germany). The filter sets applied were A, L5, and N21 (Leica Microsystems). The specimens, from which chromosome preparations were made, and the preparations themselves are stored at the Zoological Institute RAS (St. Petersburg, Russia). Adobe Photoshop CC2014 and Leica Application Suite 4.5.0 were used to process the images.

## 3. Results

The 18S rDNA probe was tested on meiotic chromosome preparations for each of the four species involved in this study.

**Figure 1:**
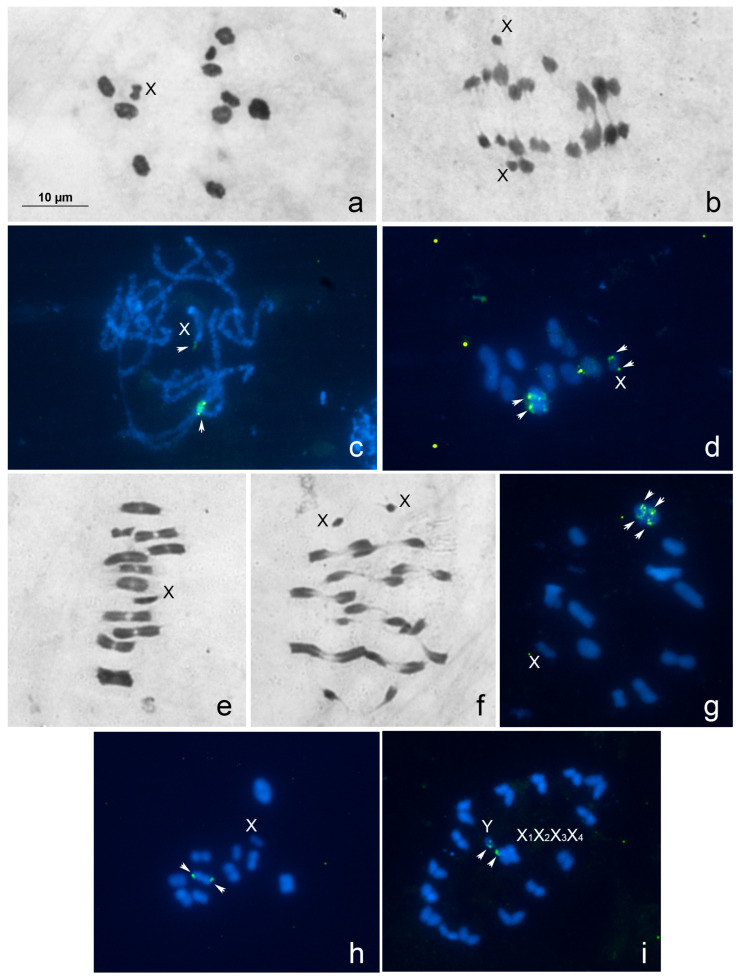
(**a**–**i**) Male meiotic chromosomes of four species of Gerromorpha after Schiff–Giemsa staining (**a**,**b**,**e**,**f**) and FISH with an 18S rDNA probe (**c**,**d**,**g**,**h**,**i**). *Limnoporus rufoscutellatus*: early metaphase I (**a**), anaphase I (**b**), prophase (pachytene stage) (**c**), metaphase I (**d**). *Aquarius paludum*: metaphase I (**e**), anaphase I (**f**), early metaphase I (**g**). *Gerris lacustris*: metaphase I (**h**). *Mesovelia furcata*: metaphase II (**i**). X and Y - the sex chromosomes; 18S rDNA green signals are shown by arrows. Bar = 10 mkm.

*Limnoporus rufoscutellatus* meioformula: n = 10AA + X; karyotype formula: 2n = 21, X(0) ♂ (Figure 1a–d).

**Karyotype.** The karyotype of *L. rufoscutellatus* was studied for the first time. In males, 10 autosomal bivalents (AA) and a univalent X chromosome were observed at the early metaphase I stage (MI) (Figure 1a), which means n = 10AA + X and, thus, 2n = 21, X(0). The autosomal bivalents make up a decreasing size series; the X chromosome is close in size to one of the small semi-bivalents and is split into chromatids. At the first anaphase (AI), the autosomal bivalents undergo normal reductional division, with the segregation of homologs, whereas the X chromosome divides equationally equatorially, segregating sister chromatids to opposite poles (Figure 1b), followed by reductional division at the second anaphase (AII). This is the so-called “sex-chromosome post-reduction”, which is typical for most Heteroptera [6].

**18S rDNA location**. FISH mapping revealed two pairs of 18S rDNA clusters on one of the larger (probably the largest) autosomal bivalents and two clusters on the X chromosome (one on each chromatid) at early prophase (pachytene stage) (Figure 1c) and MI (Figure 1d). The localization of signals in the condensed bivalents is unclear (although it is more likely to be terminal, while they were undoubtedly terminal in the X chromosome.

*Aquarius paludum* meioformula: n = 11AA + X; karyotype formula: 2n = 23, X(0) ♂ (Figure 1e–g).

**Karyotype.** The karyotype of *A. paludum* has been described repeatedly, but the published data do not always match (reviewed in [7]; see Discussion). In the present study, the males had 11 autosomal bivalents and a univalent X chromosome at MI (Figure 1e), which means n = 11AA + X and, thus, 2n = 23, X(0). The bivalents make up a decreasing size series, and the X chromosome is close in size to one of the small semi-bivalents (Figure 1e,f). At AI, autosomal bivalents undergo reductional division, whereas the X chromosome divides equationally, segregating sister chromatids to opposite poles; that is, it follows post-reductional meiosis (Figure 1f).

**18S rDNA location**. FISH mapping revealed two pairs of 18S rDNA clusters on one of the larger autosomal bivalents (probably the largest one) (Figure 1g). The localization of the signals in the condensed bivalents was unclear.

*Gerris lacustris* meioformula: n = 10AA + X; karyotype formula: 2n = 21, X(0) ♂ (Figure 1h).

**Karyotype.** The karyotype of *G. lacustris* has been described repeatedly (reviewed in [7]; see also Discussion), and our observations corroborate earlier descriptions. The males studied here had 10 autosomal bivalents and a univalent X chromosome at MI (Figure 1h), which means n = 10AA + X and, thus, 2n = 21, X(0). The autosomal bivalents make up a decreasing size series, and the X chromosome is close in size to the smallest semi-bivalent (Figure 1h).

**18S rDNA location**. FISH mapping revealed 18S rDNA clusters on one of the medium-sized autosomal bivalents, with the localization of signals in each homolog being terminal (Figure 1h).

*Mesovelia furcata* meioformula: n = 15AA + X_1_X_2_X_3_X_4_Y; karyotype formula: 2n = 35, X_1_X_2_X_3_X_4_Y ♂ (Figure 1i).

**Karyotype.** The karyotype of *M. furcata* has been described repeatedly ([39,40] see Discussion), and our observations corroborate with earlier descriptions. In the two males available in our material, the second metaphase (MII) was the only stage that allowed us to conclude about the karyotype of this species. At MII, we observed 15 autosomes, each composed of two chromatids, plus four X chromosomes and one Y chromosome, each consisting of a single chromatid (Figure 1i), meaning n = 15A + X_1_X_2_X_3_X_4_Y and, thus, 2n = 35, X_1_X_2_X_3_X_4_Y. The autosomes formed a ring, with four X chromosomes (located side by side, but each could be identified as a separate structure) and a Y chromosome in its center, forming a pseudo-multivalent X_n_Y through a process known as “touch-and-go” pairing. This metaphase II plate architecture is characteristic of true bugs in general [6].

**18S rDNA location**. FISH mapping revealed 18S rDNA clusters on one of the X chromosomes and on the Y chromosome. In both cases, the signals were located at the ends of chromosomes (Figure 1i).

## 4. Discussion

This study provides the first data on the number and chromosomal distribution of 18S rDNA loci for the infraorder Gerromorpha, as well as the first description of the karyotype of *Limnoporus rufoscutellatus*.

***Chromosome numbers and sex chromosome systems***

Of the approximately 2100 recognized species, 160 genera and eight families of the infraorder Gerromorpha (see Introduction), the number of cytogenetically studied species, genera and families has now reached 52, 21, and five (including Gerridae, Veliidae, Hebridae, Hydrometridae, and Mesoveliidae), respectively (see [7]; present study). These figures include the karyotype of *L. rufoscutellatus* (Gerridae) studied here for the first time, and three species, *Aquarius paludum* and *Gerris lacustris* (Gerridae), as well as *Mesovelia furcata* (Mesoveliidae), for which diploid numbers of chromosomes were previously known (see references above). Most of the studied species (35 of the 800 accepted) and genera (16 of the 71 recognized) belong to the most species-rich family Gerridae [6,7].

The species studied herein of Gerridae (all from the subfamily Gerrinae) displayed a variability in the 2n values. The karyotypes that we found in *L. rufoscutellatus* and *G. lacustris* (2n = 20A + X), as well as in *A. paludum (*2n = 22A + X), are currently known to be the most common (along with 2n = 18A + X) both in the family Gerridae and in the infraorder Gerromorpha as a whole [6,7,8]. It is worth noting that the diploid numbers of autosomes in the family and in the infraorder as a whole vary almost in the same range, from 18 to 30 and from 18 to 38, respectively [7]. In the small, strictly Holarctic genus *Limnoporus* (six described species), three species have previously been karyotyped, including *L. notabilis* (Drake & Hottes, 1925), *L. dissortis* (Drake & Harris, 1930), and *L. canaliculatus* (Say, 1832). It was shown that the first two species have 2n = 20A + X, as *L. rufoscutellatus* studied here for the first time, while the third species has 2n = 18A + XY.

The karyotypes of *G. lacustris* and *A. paludum* were previously described based on cytogenetic analysis [7] or (in the case of *G. lacustris*) confirmed by chromosome-level genome assembly analysis [41]. The data obtained in our study for *G. lacustris* coincide with the literature data, which are quite numerous. On the other hand, the available information about the karyotype of *A. paludum* is ambiguous (for details and discussion, see [7]). According to some data (see references [6]), this species (as *Hydrometra* Latrielle, 1797) has n = 11AA + X (the subspecies *A. p. paludum* Fabricius, 1794, which occurs throughout the Palaearctic region [7]). According to other data [42], it has n = 11AA + XY (probably the subspecies *A. p. amamiensis* Miyamoto, 1958, which occurs only in Japan [7]).

The cosmopolitan genus *Aquarius*, which is found on all continents except Antarctica, has 18 recognized species [43], and of the other three karyotyped species of this genus, *A. adelaidis* (Dojrn, 1860) has n = 11AA + X, and *A. remigis* (Say, 1832) and *A. najas* (De Geer, 1773) have n = 10AA + X (see [7]). In the predominantly Holarctic pond skater genus *Gerris* (43 species, although there are indications of several yet undescribed species [43]), at least four karyotype variants have been found in 11 species karyotyped to date: n = 10AA + X (seven species, including *G. lacustris*), n = 9AA + XY, n = 9AA + X, and n = 11AA + X (see [6]).

The *Mesovelia furcata* studied herein is the only species of the family Mesoveliidae for which karyotypic data are currently available; in all cases, it is reported to have 2n = 35, X_1_X_2_X_3_X_4_Y ([39,40], present paper). The number of autosomes, which is 30 in the diploid complement, is one of the highest known in the Gerridae, and the number of sex chromosomes (five) is uniquely high, not known in any other species of Gerromorpha.

All the species we have studied, as well as the infraorder Gerromorpha as a whole, have X(0) and XY sex chromosome systems. Statistically, the X(0) system predominates [6,7], although this predominance most likely reflects the uneven distribution of the studied species among the higher taxa of the infraorder. To get closer to solving this issue, and, accordingly, the question of the ancestral sex-determining system in Gerromorpha, we need taxonomically representative data on the infraorder. However, it is certain that multiple X chromosomes in *M. furcata* arose because of fragmentation of the X chromosome in an ancestral karyotype with the XY system (see also below). The family Mesoveliidae is widely considered to be a sister group to other semiaquatic bugs [2,3,27], and studying the chromosomal characteristics of this family may therefore be crucial for elucidating the karyotype evolution in Gerromorpha.

***Patterns of 18S rDNA distribution as revealed by FISH***

Genes for major ribosomal RNAs (rDNA) are present in multiple copies organized in tandem arrays (loci). The determination of the number and chromosomal arrangement of rDNA loci, especially 18S rDNA (part of the major 45S rDNA repeat unit that gives rise to the nucleolus), makes them important cytogenetic markers for comparative cytogenetic studies of many insect taxa [21], including Heteroptera. In this suborder of Hemiptera, seven infraorders are generally accepted [1,2]. Knowledge about the number and location of rDNA loci detected using molecular cytogenetic techniques has so far been focused on only three infraorders of true bugs—closely related, evolutionarily derived sister infraorders Cimicomorpha (more than 150 studied species) and Pentatomomorpha (about 100), as well as the infraorder Nepomorpha (about 20), which is one of the most specialized groups of Heteroptera. Representatives of these infraorders demonstrate a homogeneous distribution of 18S rDNA arrays, with one to four, while more often with one to two, sites of accumulation per haploid genome, which are typically located at terminal or interstitial regions of the chromosomes ([17,19,20,21,24,38], and references therein). The tendency to keep the number of rDNA clusters at a relatively low level is also observed in other insects and other eukaryotic organisms, which is presumably due to the existence of some limitation for a large number of rDNA loci in karyotypes [21,44,45,46]. Moreover, since haploid karyotypes with a single 45S rDNA cluster predominate in both basal and derived insect groups, this character state is considered ancestral for the class Insecta in general [21]. On the other hand, the 18S rDNA sites are located differently within karyotypes of true bugs: on autosomes, on micro-chromosomes (m-chromosomes), on sex chromosomes, or on both autosomes and sex chromosomes. The location on autosomes and, moreover, on only one pair of autosomes, seems to be prevalent and occurs in half of the species studied [20,24,38,47,48]. Many authors believe that this pattern is ancestral for the entire suborder Heteroptera [17,24,38]. However, it is worth noting that this hypothesis was proposed in the absence of data on the potentially basal lineages Enicocephalomorpha (320 species in two families [49]), Dipsocoromorpha (430 species in five families [50]), and Gerromorpha (2100 species in eight families, most of them in Gerridae [2]). The only information currently available about these infraorders concerns *Limnogonus aduncus* Drake & Harris, 1933 (Gerridae), in which active NORs (Nucleolus Organizing Regions, i.e., the chromosome sites where repetitive clusters of rRNA genes are located) were detected by the silver nitrate impregnation technique (Ag-NOR banding) on one pair of autosomes [51]. However, the number of transcriptionally active NORs (detected by AgNO_3_) does not necessarily correlate with the number of rDNA loci (detected by FISH) in a given species; moreover, some nucleoli can be formed in chromosome regions where FISH does not detect the presence of rDNA, suggesting the existence of cryptic rDNA loci [44]. This can be explained by the fact that the NORs detected by conventional silver staining indicate the presence of functional ribosomal genes, whereas FISH with specific rDNA probes is a highly selective method for staining interphase nucleoli and NORs on mitotic and meiotic chromosomes that were transcriptionally active during the preceding interphase. This technique allows for the precise localization of ribosomal genes. The size of the silver signal on chromosomes is proportional to the previous transcriptional level of the NOR. In contrast, FISH with rDNA probes directly detects the location of ribosomal cistrons regardless of their activity status, and the size of the FISH signals for a particular rDNA cluster is proportional to the number of ribosomal cistrons it contains [21].

Our study is thus the first attempt to determine the number and location of 18S rDNA clusters in chromosomes of Gerromorpha using FISH. Among the semiaquatic bug species investigated here, chromosomal mapping revealed one (in *G. lacustris* and *A. paludum*) or two (in *L. rufoscutellatus* and *M. furcata*) rDNA clusters per haploid genome, as found in most true bugs and in most other insects studied to date (see above). In the first two species, these sites are located on one pair of autosomes—one of the medium-sized and the largest, respectively; in *L. rufoscutellatus*—on one pair of autosomes (probably the largest) and on the X chromosome; and in *M. furcata*—on two sex chromosomes, one of the four X chromosomes and the Y. All these location patterns are among the four currently known in true bugs ([17,19,20,21,24,38], and references therein). In the rDNA-bearing bivalent of *G. lacustris* and in the sex chromosomes of *L. rufoscutellatus* and *M. furcata*, the FISH signals were located distally terminally. However, their localization in the rDNA-bearing autosomal bivalents of *A. paludum* and *L. rufoscutellatus* remained questionable.

It is noteworthy that the location of the major rDNA loci on one of several X chromosomes in species with Xn(0) or XnY systems has previously been noted in some other true bugs. Examples include species of the families Cimicidae [52,53] and Reduviidae [20,54,55,56,57,58,59] of the infraorder Cimicomorpha. These observations may indicate fragmentation of the ancestral X chromosome, with one of the newly formed X chromosomes retaining a cluster of ribosomal genes, which apparently also occurred in the evolution of *M. furcata* from our study. This assumption is consistent with the existing hypothesis that multiple sex chromosomes in Heteroptera could arise from the fragmentation of the X or (less often) Y chromosomes in a karyotype that originally possessed a simple sex chromosome system ([6,8,60], and references therein). Thus, the mapped 18S rDNA cluster serves as a cytogenetic marker of chromosomal rearrangements, which is especially important in the case of holokinetic chromosomes, which lack a localized centromere as a natural landmark.

## 5. Conclusions

In recent decades, a significant amount of data has been accumulated on the number and chromosomal distribution of rDNA arrays in true bugs. However, prior to our study, such data were only available for the infraorders Nepomorpha, Cimicomorpha, and Pentatomomorpha. The infraorder Gerromorpha comprises about 2100 species, which are currently subdivided into 160 genera and eight families, but only four species, three genera and two families currently have the rDNA-FISH data. Even based on taxonomically limited material, it can be assumed that gerromorphs are characterized by conservatism in the number of 18S rDNA loci (one or two per haploid genome), as well as the dynamic evolution of these loci in terms of their chromosomal distribution (only on autosomes, on autosomes and on the X chromosome, and only on sex chromosomes). These observations are consistent with the trends identified earlier in each of the three other infraorders studied in this regard. However, many issues remain unresolved. In particular, it is unknown which location of the rDNA clusters is ancestral in Gerromorpha and what mechanism (or mechanisms) underlies their movement during the evolution of the infraorder. We believe that future rDNA-FISH studies involving a larger number of species, especially those from basal taxa, will lead to new discoveries in this field.

## Figures and Tables

**Table 1:** Species studied, number of samples, dates and places of collection.

Species	Number of Specimens Studied, Data and Collection Sites
*Aquarius paludum* (Fabricius, 1794)	3 males, 3 August 2025, 15 km N Voronezh, Usmanka River, Russia (51°48′56.3″ N 39°22′53.2″ E)
*Gerris lacustris* (Linnaeus, 1758)	2. males, 12 June 2025, Leningrad Region, vic. of village Ploskoe, Luga River, Russia (58°48′53.2″ N 29°58′14.0″ E) 2 males, 3 May 2019, vic. of Goce Delchev, River of Mesta, Bulgaria (41°34′23.4″ N 23°43′29.1″ E)
*Limnoporus rufoscutellatus* (Latreille, 1807)	2 males, 28 June 2025, Murmansk Region, vic. of village Olenitsa, desalinated temporary reservoir, Russia (66°27′15.0″ N 35°18′07.2″ E)
*Mesovelia furcata* Mulsant & Rey, 1852	2 males, 3 May 2019, vic. of Goce Delchev, River of Mesta, Bulgaria (41°34′23.4″ N 23°43′29.1″ E)

## Data Availability

The original contributions presented in this study are included in this article. Further inquiries can be directed to the corresponding authors.
